# Chest Pain in a Psychiatric Patient Due to Clozapine-Induced Myopericarditis

**DOI:** 10.1155/2021/6067652

**Published:** 2021-12-24

**Authors:** James L. Laws, Esther Kim, Rebecca Hung, JoAnn Lindenfeld, Richa Gupta

**Affiliations:** ^1^Department of Internal Medicine, Vanderbilt University Medical Center, Nashville, Tennessee, USA; ^2^Department of Cardiovascular Medicine, Vanderbilt University Medical Center, Nashville, Tennessee, USA; ^3^Department of Cardiovascular Medicine, MedStar Washington Hospital Center, Washington, DC, USA

## Abstract

Drug-induced myocarditis is a rare, but underrecognized complication of clozapine therapy for schizophrenia. We present a case of clozapine-induced myocarditis with recovery of cardiac function after drug cessation and summarize the literature to highlight the variable presentation of this condition.

## 1. Case Presentation

A 29-year-old man presented from a psychiatric hospital with 2 days of sudden onset, progressive chest pain, and troponin elevation after recently initiating clozapine therapy.

The patient had a history of alcohol abuse and treatment-resistant schizoaffective disorder with multiple suicide attempts. After intentional ingestion of acetaminophen and lithium, he was involuntarily hospitalized for intensive treatment, including initiation of clozapine therapy.

**Figure 1:**
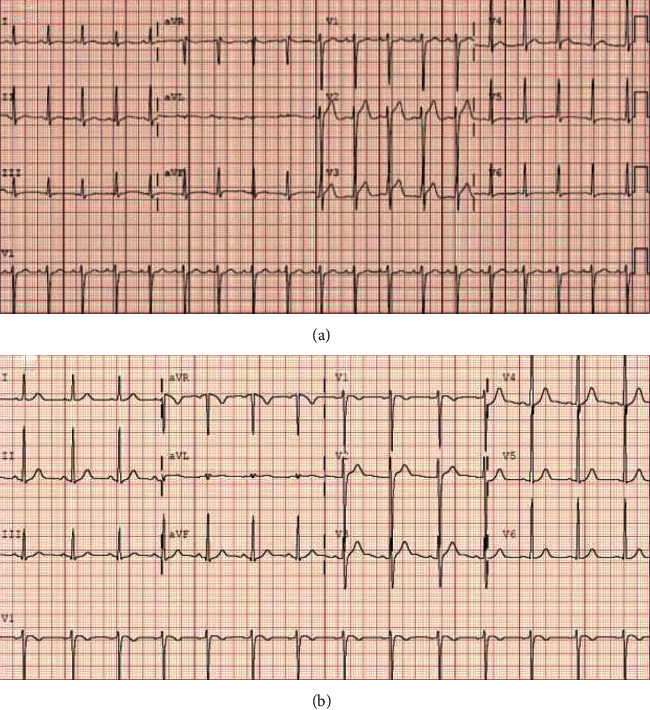
Electrocardiogram on presentation (a) showed sinus tachycardia (heart rate 111 beats per minute), ST elevations most prominent in leads V2-V3 with inferolateral T wave flattening. Baseline EKG (b) showing less prominent ST elevations in V2-V3 consistent with early repolarization without inferolateral T wave flattening.

The differential diagnosis included acute coronary syndrome, myopericarditis from viral or drug-induced etiology, pulmonary embolism, and pneumonia. The patient first noted substernal chest pain worse with deep inspiration 8 days after clozapine initiation. Symptoms worsened over the following two days. Electrocardiogram (EKG) demonstrated ST elevations most prominent in V2-V3 (Figure 1). Serum labs were notable for leukocytosis and elevated troponin, brain natriuretic peptide, and inflammatory markers (Table 1).

**Figure 2:**
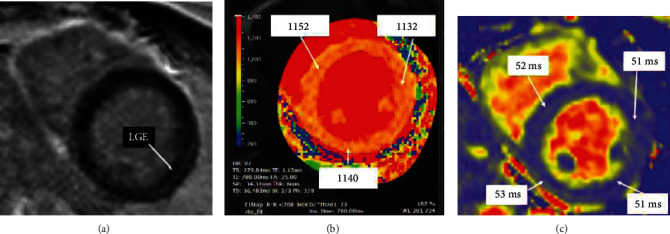
Tissue characterization on presenting cardiac MRI. Subtle stripe of epicardial LGE noted in the lateral wall (a). The native T1 relaxation time is diffusely elevated throughout the myocardium (ULN for this 1.5 Tesla magnet is 1010 ms), suggestive of diffuse inflammation (b). The native T2 relaxation time is mildly elevated throughout the myocardium and highest in the inferior wall (ULN: 50 ms), suggestive of edema (c). Abbreviations: LGE: late gadolinium enhancement; ULN: upper limit of normal.

Chest pain persisted, and he was transferred to the cardiology service. Cardiovascular exam was notable for tachycardia without murmurs or rubs. He had no peripheral edema or elevated jugular venous pressure. Lungs were clear. Cardiac magnetic resonance (CMR) imaging showed global hypokinesis with a left ventricular (LV) ejection fraction (EF) of 45% (Figure S1) and subtle late gadolinium enhancement of the epicardial lateral wall and lateral pericardium with abnormal parametric mapping (Figure 2) consistent with myopericarditis. Respiratory viral panel was negative. Clozapine was discontinued. Metoprolol was initiated for impaired LV function with colchicine and ibuprofen for pericarditis with prompt symptomatic relief. Repeat EKG showed resolution of ST-elevation and no arrhythmic events on telemetry. He was discharged back to the psychiatric hospital with planned cardiology follow-up for echocardiogram and repeat laboratory testing.

## 2. Discussion

Myocarditis was suspected based on a clinical syndrome of chest pain and elevated troponin in the setting of a known causative medication and low pretest probability for coronary artery disease. CMR confirmed the diagnosis by the updated Lake Louise Criteria [1]. This case meets the ESC definition of myocarditis, as a clinical syndrome supported by laboratory and imaging findings [2]. Endomyocardial biopsy (EMB), long considered the gold standard for diagnosis, is not indicated unless histology would alter treatment as with certain fulminant subtypes like giant cell myocarditis.

Clozapine-induced myocarditis (CIM) is a drug-related myocarditis with variable presentation ranging from mild symptoms to fulminant myocarditis. The mechanism of toxicity to cardiomyocytes is unclear. In animal models, clozapine induces serum catecholamine excess that causes cardiac inflammation and myocyte apoptosis through tumor necrosis factor-alpha-mediated pathways [3]. At autopsy, histologic specimens from CIM patients have shown either lymphocytic infiltrate resulting from cytotoxic-mediated inflammation or eosinophilic infiltrate consistent with a hypersensitivity myocarditis, suggesting multiple mechanisms of injury [4].

The true incidence of CIM is difficult to ascertain due to underdiagnosis of subclinical or mild presentations and may be underrecognized; thus, a high index of suspicion is required [5]. Shortness of breath and tachycardia (67% and 58%, respectively) are the most common symptoms, though nonspecific [6]. In one cohort, 13% of patients ultimately diagnosed with CIM had normal cardiac biomarkers. Ronaldson and colleagues proposed a monitoring program based on review of suspected cases using serial troponin and C-reactive protein measurements over a 4-week initiation period [7]. No prospectively validated algorithms exist, however, and monitoring practices vary by institution. While some patients may present with a classic syndrome of myocarditis prompting additional evaluation, sudden cardiac death may be the initial presentation, highlighting the importance of monitoring [8].

Cardiovascular complications resulting from antipsychotic medications are not limited to myocarditis. Patients with schizophrenia treated with antipsychotics are at higher risk of sudden unexplained death than the general population [8]. Cardiovascular, pulmonary, and hematologic causes have been implicated with high prevalence of myocardial infarction (53%), myocarditis (6%), and a substantial proportion remaining unexplained (12%) [9]. Early recognition of angina and myocardial injury should prompt further evaluation and intervention crucial to preventing sudden cardiac death.

When CIM is suspected, cardiology consultation should be obtained with inpatient monitoring for arrhythmia and heart failure. Aside from drug withdrawal, no specific treatment for CIM has been identified. Patients with LV systolic dysfunction warrant guideline-directed medical therapy (GDMT) in the absence of contraindications. Close cardiology follow-up and repeat echocardiography after clozapine cessation and initiation of GDMT should be individualized.

Intriguingly, published literature reports cases of clozapine reinitiation in patients with severe schizophrenia refractory to alternate antipsychotics despite prior drug withdrawal due to CIM. Rechallenge with clozapine can be successfully achieved with intensive monitoring with swift discontinuation of the drug for any sign of myocardial injury [10]. In one case, serial CMR was used to monitor for disease recurrence and allowed the patient to safely remain on clozapine therapy [11]. Thus, individualized shared decision-making between patient, cardiology, and psychiatry is the key to optimize both cardiac and psychiatric outcome for these patients.

## 3. Follow-Up

The patient was not reinitiated on clozapine and instead was treated with lithium and electroconvulsive therapy. Repeat echocardiography 2 weeks later demonstrated recovery of LV function (Figure S2).

## 4. Conclusions

Patients with schizoaffective disorders treated with antipsychotics are at higher risk for sudden unexplained death. CIM is a rare but likely an underrecognized complication of clozapine therapy and a mechanism of sudden cardiac death. Treating psychiatrists and consulting cardiologists must maintain high index of suspicion for CIM with withdrawal of clozapine in the acute setting to reduce poor outcomes. In the long term, care must be patient-centered with consideration of underlying psychiatric and cardiovascular disease severity.

## 5. Learning Objectives

- To recognize the clinical presentation of drug-induced myocarditis, associated with the use of clozapine
- To illustrate the role of noninvasive testing in diagnosis of clozapine-induced myocarditis
- To understand the importance of a multidisciplinary approach involving both psychiatry and cardiology specialists in managing clozapine-induced myocarditis

## Figures and Tables

**(a):** 

Lab	At presentation	7 d follow-up
White blood cell count (cells/*μ*L)	14.9	—
Absolute eosinophils (cells/*μ*L)	0.06	—
C-reactive protein (*μ*g/mL)	173.0	5.1
Erythrocyte sedimentation rate (mm/hr)	64	5
Brain natriuretic peptide (pg/mL)	311	33

**(b):** 

	Presentation	4 h	Discharge (2 d)
Troponin-I (ng/mL, normal<0.03)	0.49	0.37	0.05

## Data Availability

The data supporting this case report are from previously reported studies and datasets, which have been cited. The images used are available to view at https://drive.google.com/drive/folders/1casDU91KCqFa8M.

## Supplementary Materials

Supplementary Materials Figure S1: Left ventricular function on presenting cardiac MRI. Four chamber (a) and axial cine stacks (b) obtained at presentation showed left ventricular ejection fraction of 45% by volumetric assessment with mild inferolateral hypokinesis and a trivial pericardial effusion. Figure S2: Follow-up transthoracic echocardiogram. One week after clozapine cessation and initiation of heart failure therapies, follow-up echocardiogram showed normalization of left ventricular function with no wall motion abnormalities on apical 4 chamber (a) and short axis (b) cines.
